# Incompetent lip seal and nail biting as risk factors for malocclusion in Japanese preschool children aged 3–6 years

**DOI:** 10.1186/s12887-023-04366-7

**Published:** 2023-10-26

**Authors:** Masatoshi Otsugu, Yumi Sasaki, Yusuke Mikasa, Maika Kadono, Hidekazu Sasaki, Takafumi Kato, Kazuhiko Nakano

**Affiliations:** 1 Department of Pediatric Dentistry, Osaka University Graduate School of Dentistry, 1-8 Yamada-oka, Suita, 565-0871 Osaka Japan; 2 Department of Oral Physiology, Osaka University Graduate School of Dentistry, Osaka, Japan

**Keywords:** Preschool children, Malocclusion, Oral habits, Incompetent lip seal, Nail biting

## Abstract

**Background:**

Malocclusion is a multifactorial condition associated with genetic and environmental factors. The purpose of this study was to investigate the prevalence of occlusal traits, oral habits, and nose and throat conditions by age and to assess the association between malocclusion and its environmental factors in Japanese preschool children.

**Methods:**

A total of 503 Japanese children (258 boys and 245 girls aged 3–6 years) were recruited. Occlusal traits were assessed visually to record sagittal, vertical, and transverse malocclusion, and space discrepancies. Lip seal was recorded by an examiner, and oral habits (finger sucking, lip sucking or lip biting, nail biting, chin resting on a hand) and nose and throat conditions (tendency for nasal obstruction, allergic rhinitis, palatine tonsil hypertrophy) were assessed by a questionnaire completed by the parents. The prevalence of each item was calculated, and binary logistic regression was used to examine the factors related to malocclusion.

**Results:**

62.0% of preschool children in the present study exhibited malocclusion, and 27.8% exhibited incompetent lip seal. Nail biting was the most frequent oral habit with a prevalence of 18.9%. Nasal obstruction was recorded in 30.4% of children. The results of binary logistic regression showed that incompetent lip seal was significantly related to malocclusion, and that nail biting was significantly negatively related.

**Conclusions:**

Incompetent lip seal is significantly associated with malocclusion, but nail biting may not necessarily be a deleterious habit for the occlusion in Japanese preschool children.

## Background

Malocclusion is a growth and development deviation, mainly of the muscles and jaw bones, during childhood and adolescence [1]. It is characterized by the presence of misaligned teeth and/or maxillary and mandibular discrepancies [2]. The prevalence of malocclusion in the primary dentition or in preschool children has been reported to range from 45.5 to 83.9% based on the different criteria used throughout the world [2–12]. It is considered that malocclusion in the primary dentition is a precursor for malocclusion in the permanent dentition, and some malocclusions tend to persist during the development of the occlusion [5, 13–15]. Therefore, it is beneficial to discuss the causes of malocclusion in early childhood.

Malocclusion is thought to be a multifactorial condition associated with genetic and environmental factors [16, 17]. Major environmental factors affecting malocclusion include thumb sucking, nail biting (onychophagia), and tongue thrusting of early childhood, which are repetitive oral behaviors [1–3, 5, 9, 11, 16, 17]. Oral habits can interfere not only with the position of the teeth, but also with normal skeletal growth [18]. Therefore, when these problems are found in association with malocclusion, they will influence the prognosis and should be eliminated to ensure a functional environment adequate for physiological growth [1, 18].

Thumb sucking is a common oral habit reported in 5–25% of children and occurs almost universally in early infancy [2, 19–22]. Prolonged thumb sucking can lead to an imbalance between external and internal muscles in the maxillofacial region, resulting in an increased prevalence of certain malocclusions such as excessive overjet, anterior open bite, and posterior crossbite [1, 3, 5, 16, 18, 21]. However, the presence of thumb sucking does not necessarily lead to malocclusion, although the intensity, frequency, and duration are thought to be associated [18, 23]. Nail biting is another common oral habit, and its prevalence reported to be approximately 15–25% [19, 20, 22]. Evidence that nail biting leads to malocclusion remains ambiguous [24]. Additionally, developmental factors, such as impaired nasal breathing, palatine tonsil hypertrophy, and incompetent lip seal (ILS) are considered to be important environmental factors in the etiology of malocclusion [2, 25–28]. In particular, a recent study found that approximately 30% of Japanese children exhibited ILS, and thus the impact of ILS on malocclusion warrants further investigation because of its high frequency [27]. However, little information is available about the prevalence of malocclusion and the above environmental factors, and their influence on preschool children, especially in Japanese.

The purpose of this study was to investigate the prevalence of occlusal traits, oral habits, and nose and throat conditions by age and to assess the association between malocclusion and environmental factors in Japanese preschool children.

## Methods

### Ethics statement

This study was conducted with full adherence to the Declaration of Helsinki. The study protocol was approved by the Ethics Committee of Osaka University Graduate School of Dentistry (approval number H29-E39). Written informed consent was obtained from the parents of all participants, and verbal agreement for participation was obtained from the children.

### Participants

A total of 553 Japanese children from one kindergarten in Osaka City were invited to participate in the study. The staff of this school distributed letters outlining the purpose of the study and consent forms to the guardians of all the children. The inclusion criteria were as follows: age 3–6 years; normal language comprehension; no uncooperative behavior; no reported systemic disease. The exclusion criteria included lack of informed consent or willingness to participate in the study. A total of 503 healthy children (258 boys and 245 girls aged 3–6 years) were recruited for the study.

### Dental occlusal assessment

Dental occlusal assessment was performed as follows based on the method proposed by the Japanese Society of Pediatric Dentistry (2015, Tokyo, Japan), with an additional assessment, as well as our previous study [29]. In the assessment of sagittal malocclusion, anterior crossbite was defined as negative overjet of at least one incisor (< 3 incisors: mild; ≥ 3: moderate), and excessive overjet was defined as excessive increased overjet (≥ 4 mm). In the assessment of vertical malocclusion, deep overbite was defined as excessive increased overbite (≥ 4 mm), and anterior open bite was defined as a lack of vertical contact between the upper and lower teeth in the anterior region. In the assessment of transverse malocclusion, posterior crossbite was defined as the buccal cusp of at least one upper primary molar tooth occluding lingually with the lingual cusp of the corresponding lower tooth, and scissors bite was defined as the lingual cusp of at least one upper primary molar tooth occluding lingually with the buccal cusp of the corresponding lower tooth [30]. In the assessment of space discrepancies, crowding was defined as upper primary incisor teeth or lower primary incisor teeth overlapping even slightly. Children who exhibited at least one of these conditions were classified as having malocclusion.

### Dentition traits assessment (midline deviation, unspaced dentitions)

Apart from the above criteria for malocclusion, dentition traits were assessed. Mandibular midline deviation against the maxillary midline to left or right was defined as midline deviation by at least 1 mm [3]. Additionally, unspaced dentitions were defined as dentitions with neither primate spaces nor developmental spaces in both the maxilla and mandible.

### Lip seal

Lip seal was recorded by observation of the child’s appearance in the resting position as they entered the room before being aware of the pending examination, and was defined as ‘competent’, or ‘incompetent’ if the lips were naturally closed or apart, respectively [31, 32]. ILS was also defined as the condition in which any part of the crown of the maxillary incisors remained visible in other studies [33, 34]. Therefore, the condition was defined as severe ILS, in the present study.

Dental examinations were performed with the aid of a penlight, mouth mirror, and metal millimeter ruler. All examinations for dental occlusion, dentition traits, and lip seal were performed by one pediatric dentist in the Department of Pediatric Dentistry of Osaka University.

### Questionnaire for parents about children’s oral habits and nose and throat conditions

The questionnaire for parents consisted of seven questions about their children’s oral habits (finger sucking, lip sucking or lip biting, nail biting, chin resting on a hand) and nose and throat conditions (tendency for nasal obstruction, allergic rhinitis, palatine tonsil hypertrophy). All questions were yes or no items as per the protocol. The staff of the school gave the parents the questionnaires and collected them following completion. All submitted surveys remained completely anonymous and did not include any personal information that might identify the respondent or their child.

### Statistical analysis

The prevalence of malocclusion and the different types of malocclusions, dentitions, and nose and throat conditions was analyzed using the chi-squared test by sex and the Cochran-Armitage test for age trend. For mandibular midline deviation, the chi-squared test was applied between the shift to right and left. In addition, the chi-squared test or Fisher’s exact test was applied where appropriate for comparisons between normal occlusion and malocclusion [35, 36]. Furthermore, binary logistic regression with malocclusion as the dependent variable was done to calculate odds ratios and 95% confidence intervals after controlling simultaneously for potential confounding factors. Independent variables in the model were age, sex, incompetent lip seal, finger sucking, lip sucking or lip biting, nail biting, chin resting on a hand, stuffy nose, and palatine tonsil hypertrophy. The fit of the data to the model was tested using the Hosmer-Lemeshow test and multicollinearity was assessed by using the variance inflation factor (VIF).

The sample size was based on an estimated 1 variable for logistic regression analysis. For reliable analysis, we required at least 10 events of the primary outcome measure per variable, that is, 90 events for 9 variables [37]. Given a prevalence of malocclusion in Japanese preschool children of 53.5% [29], the required sample size was 169 or more. All data were analyzed using IBM SPSS statistics version 28.0.1.0. (IBM Japan, Tokyo, Japan). Statistical significance was set at the *P* < 0.05 level.

## Results

### Prevalence of malocclusion

62.0% (312 out of 503) of the children in the present study showed some type of malocclusion. No significant age trends were found in the prevalence of malocclusion. Table 1 summarizes the prevalence of each type of malocclusion in the study population. The most frequent types were excessive overjet with a prevalence of 27.8%, followed by deep overbite (23.1%), crowding (11.5%), anterior crossbite (9.5%), and anterior open bite (7.2%). No children exhibited transverse malocclusion, such as posterior crossbite or scissors bite in the present study. Additionally, a total of 86 children exhibited two or more types of malocclusions (17.2%). No age trends were found in any type of malocclusion in the Cochran-Armitage test for trend, but crowding showed significantly higher frequency in girls than boys (*P* < 0.01).

**Table 1:** Prevalence of each type of malocclusion in preschool children aged 3–6 years

	3 years old	4 years old	5 years old	6 years old	Total
	(n = 119)	(n = 171)	(n = 146)	(n = 67)	(n = 503)
**Sagittal malocclusion**	42.0%	33.3%	37.7%	38.8%	37.4%
Anterior crossbite (AC)	11.8%	7.0%	10.9%	9.0%	9.5%
(mild/moderate)	(3.4%/8.4%)	(1.8%/5.3%)	(3.4%/7.5%)	(1.5%/7.5%)	(2.6%/7.0%)
Excessive Overjet (E)	30.3%	26.3%	26.7%	29.9%	27.8%
**Vertical malocclusion**	29.4%	33.3%	26.0%	32.8%	30.2%
Deep overbite (D)	23.5%	26.3%	18.5%	23.9%	23.1%
Anterior open bite (AO)	5.9%	7.0%	7.5%	9.0%	7.2%
**Transversal malocclusion**	0%	0%	0%	0%	0%
Posterior crossbite	0%	0%	0%	0%	0%
Scissors bite	0%	0%	0%	0%	0%
**Space discrepancies**	16.0%	7.6%	12.3%	11.9%	11.5%
Crowding (C)	16.0%	7.6%	12.3%	11.9%	11.5%**
**Multiple types of malocclusion**	20.2%	15.8%	16.4%	16.4%	17.2%
**Total**	67.2%	58.5%	59.6%	67.2%	62.0%

**: *P* < 0.01 (Chi-squared test between boys and girls) (boys: 7.8%; girls: 15.5%)

### Prevalence of tooth and dentition traits

Mandibular midline deviation was observed in 40.4% of the children (203 out of 503) and shift to the right showed significantly higher frequency than shift to the left (*P* < 0.01) (Table 2). Additionally, unspaced dentitions were in observed in 33.0% (166 out of 503), with a significantly higher frequency in girls than boys (*P* < 0.01).

**Table 2:** Prevalence of dentition traits in preschool children aged 3–6 years

	3 years old	4 years old	5 years old	6 years old	Total
	(n = 119)	(n = 171)	(n = 146)	(n = 67)	(n = 503)
**Mandibular midline deviation**	35.3%	43.3%	43.2%	35.8%	40.4%
right	17.6%	28.7%	25.3%	22.4%	24.3%^a^
left	17.6%	14.6%	17.8%	13.4%	16.1%
**Unspaced dentitions**	40.3%	31.6%	31.5%	26.9%	33.0%^b^

a: *P* < 0.01 (Chi-squared test between right and left)

b: *P* < 0.01 (Chi-squared test between boys and girls) (boys: 26.7%; girls: 39.6%)

### Oral habits and nose and throat conditions

Table 3 shows oral habits and nose and throat conditions of the children. 27.8% of children exhibited ILS and approximately 15.7% of them were severe. No age trends were found for prevalence. Among oral habits, nail biting (18.9%) was the most frequent, followed by finger sucking (7.8%), chin resting on a hand (3.2%), and lip sucking or biting (3.0%). Chin resting on a hand showed a significant increasing trend with age from 3 years to 6 years (*P* < 0.001). Regarding nasal diseases, 30.4% of children tended to experience nasal obstruction, and 17.1% had allergic rhinitis. Allergic rhinitis showed a significant increasing trend with age from 3 years to 6 years (*P* < 0.001). Additionally, 3.6% had palatine tonsil hypertrophy. Furthermore, chin resting on a hand (*P* < 0.05), allergic rhinitis (*P* < 0.05), and palatine tonsil hypertrophy (*P* < 0.01) recorded significantly higher frequency in boys than girls.

**Table 3:** Oral habits and nose and throat conditions in preschool children aged 3–6 years

	3 years old	4 years old	5 years old	6 years old	Total
	(n = 119)	(n = 171)	(n = 146)	(n = 67)	(n = 503)
**Incompetent lip seal**	26.1%	32.7%	25.3%	23.9%	27.8%
severe	12.6%	17.5%	15.8%	16.4%	15.7%
**Habit**					
Nail biting	16.0%	22.2%	17.1%	19.4%	18.9%
Finger sucking	7.6%	8.2%	6.8%	9.0%	7.8%
Lip sucking or lip biting	5.0%	1.2%	4.1%	1.5%	3.0%
Chin resting on a hand	0.8%	0.6%	6.2%	7.5%	3.2%^a,b^
**Nose and throat condition**					
Nasal obstruction	30.3%	34.5%	27.4%	26.9%	30.4%
Allergic rhinitis	5.9%	17.0%	18.5%	34.3%	17.1%^a,b^
Palatine tonsil hypertrophy	1.7%	4.1%	3.4%	6.0%	3.6%^c^

a: *P* < 0.001 (Cochran-Armitage test for trend)

b: *P* < 0.05 (Chi-squared test between boys and girls)

Chin resting on a hand (boys: 5.0%; girls: 1.2%)

Allergic rhinitis (boys: 20.9%; girls: 13.1%)

c: *P* < 0.01 (Chi-squared test between boys and girls) (boys: 5.8%; girls: 1.2%)

### Related factors and characteristics of malocclusion

Table 4 shows the related factors and characteristics of malocclusion compared with normal occlusion. ILS (*P* < 0.01), especially severe ILS (*P* < 0.001), palatine tonsil hypertrophy (*P* < 0.05), and unspaced dentitions (*P* < 0.001) recorded significantly higher frequency in malocclusion than in normal occlusion, while nail biting (*P* < 0.05) recorded significantly lower frequency. The results of binary logistic regression showed that ILS (OR = 1.838; 95% CI: 1.189–2.841, *P* < 0.01) and nail biting (OR = 0.625; 95% CI: 0.393–0.994, *P* < 0.05) were significantly associated with malocclusion after controlling simultaneously for potential confounding factors (Table 5). In the regression model, the Hosmer-Lemeshow showed a good fit (*P* = 0.707), and VIF showed the absence of multicollinearity for each independent variable (< 4.0).

**Table 4:** Related factors and characteristics of malocclusion compared with normal occlusion

	Normal	Malocclusion	*P*
	(n = 191)	(n = 312)	
Boy ratio	51.8%	51.0%	n.s.
Incompetent lip seal	19.9%	32.7%	< 0.01
Severe incompetent lip seal	8.9%	19.9%	< 0.001
Nail biting	23.6%	16.0%	< 0.05
Finger sucking	5.2%	9.3%	n.s.†
Lip sucking or lip biting	2.1%	3.5%	n.s.†
Chin resting on a hand	4.2%	3.5%	n.s.†
Nasal obstruction	25.1%	32.7%	n.s.
Allergic rhinitis	17.3%	17.0%	n.s.
Palatine tonsil hypertrophy	1.0%	5.1%	< 0.05†
Mandibular midline deviation	35.1%	43.6%	n.s.
Unspaced dentitions	23.6%	38.8%	< 0.001

†: Fisher’s exact test

n.s.: not significant

**Table 5:** Binary logistic regression analysis of related factors and malocclusion

	B	SE	Adjusted OR (95% CI)	*P*
Age	-0.025	0.097	0.975 (0.806–1.180)	0.797
Sex	-0.051	0.192	0.950 (0.653–1.383)	0.790
Incompetent lip seal	0.609	0.222	1.838 (1.189–2.841)	0.006
Nail biting	-0.470	0.237	0.625 (0.393–0.994)	0.047
Finger sucking	0.548	0.388	1.730 (0.809–3.701)	0.158
Lip sucking or lip biting	0.199	0.623	1.220 (0.360–4.139)	0.749
Chin resting on a hand	0.264	0.587	1.302 (0.412–4.115)	0.653
Nasal obstruction	0.352	0.211	1.422 (0.940–2.152)	0.095
Palatine tonsil hypertrophy	1.449	0.771	4.260 (0.940-19.308)	0.060

Adjusted by age, sex, incompetent lip seal, finger sucking, lip sucking or lip biting, nail biting, chin resting on a hand, stuffy nose, and palatine tonsil hypertrophy

## Discussion

### Prevalence of malocclusion

In the present study, 62.0% of the preschool children showed some type of malocclusion. The prevalence of malocclusion in this general age group is reported to be diverse throughout the world, ranging from 45.5 to 83.9% [2–12, 29]. In our previous study, we found that 53.5% of Japanese children aged 4–6 years showed some type of malocclusion [29], and the higher frequency in the present study may have arisen from the inclusion of 3-year-old children. Additionally, excessive overjet and deep overbite recorded the highest prevalence in the present study, which was consistent with our previous study and previous studies globally (excessive overjet: 10.2–46.1%, deep overbite: 6.05–41.5%) [2–12, 29]. However, no children exhibited transverse malocclusion in the present study, although several children had a lateral edge-to-edge bite, which was not included in the malocclusion criteria. These findings were consistent with our previous study [29]. Although Asians generally have a low prevalence of posterior crossbite and scissors bite [6, 30], further investigation is necessary to clarify the detailed prevalence in larger samples.

### Dentition traits

In the present study, apart from the criteria for malocclusion, mandibular midline deviation and unspaced dentitions were investigated as dentition traits. Mandibular midline deviation was observed in 40.4% of children, which was higher than in previous studies (21.9–26.6%) [2, 3, 6], and was not related to the four oral habits in the present study (data not shown). Interestingly, right-side deviation recorded significantly higher frequency than left-side deviation. Midline deviation is generally caused by lateral mandibular deviation related to posterior crossbite, tipping and/or drifting of the incisors, arch asymmetry, or any combination of these factors [38]. Although the cause of the present findings remains unclear, right-handedness for tool use is frequent in Japanese people [39], and the habitual chewing side may tend to become the same as the handedness as the frequency of eating tool use increases. Unspaced dentitions were in observed in about 35% of children. In most previous studies, space analysis in the dentition was divided into the maxilla and the mandible, or primate space and developmental space, making comparison with the present study difficult [40–42]; however, unspaced dentitions in both the maxilla and mandible were more prevalent in the present study than in a previous study. (18.1%) [43]. This may be related to ethnic differences or may be evidence of an increasing trend. Additionally, unspaced dentitions were found to have significantly higher frequency in girls than boys (*P* < 0.01). This tendency was consistent with previous studies [40, 41], and is considered to be universal.

### Oral habits and nose and throat conditions

27.8% of children exhibited ILS in the present study, which was similar to a previous study in Japan [27]. However, ILS did not show a significant increasing trend with age in the present study, which was inconsistent with the previously mentioned study [27], probably because of the limited age range (3–6 years). The prevalence of nail biting (18.9%), finger sucking (7.8%), and lip sucking or biting (3.0%) was also similar to previous studies (nail biting: 15.2–23.0%, finger sucking: 5.6–25.0%, lip sucking or biting: 4.7–5.6%) [2, 19–22], and no significant age trend was observed in the present study. It is thought that the prevalence of these oral habits remains constant in the preschool years, and then decreases with age [44]. However, chin resting on a hand recorded significantly higher frequency in boys than in girls (*P* < 0.05), with a significantly increasing trend with age from 3 years to 6 years. (*P* < 0.001). Chin resting on a hand is considered to exert lateral pressure on the jaws [45, 46]. The increasing trend in this habit may be related to an increase in sedentary behavior among Japanese children depending on age.

Approximately 30% of children tended to have nasal obstruction, and 17% had been diagnosed with allergic rhinitis in otolaryngology and pediatric clinics. Additionally, allergic rhinitis occurred significantly more frequently in boys than girls (*P* < 0.05), with a significantly increasing trend with age from 3 years to 6 years (*P* < 0.001). Allergic rhinitis has been reported to show a moderate increasing trend and be more prevalent among boys during childhood [47, 48]. Although nasal obstruction is a symptom of allergic rhinitis, it can be associated with various diseases, such as non-allergic rhinitis, chronic sinusitis, and severe septal deviation, resulting in a higher frequency than allergic rhinitis [49]. Furthermore, 3.6% of the children had been diagnosed with palatine tonsil hypertrophy in otolaryngology and pediatric clinics, with a significantly higher frequency in boys than girls (*P* < 0.01). Tonsil size significantly increases during the first 3 years of life, with only a moderate and not significant increase from 3 years to 12 years [50]. However, the lingual tonsils comprise Waldeyer’s ring with the palatine tonsils, and their hypertrophy is more frequently observed in patients with allergic rhinitis; therefore, palatine tonsil hypertrophy, as well as allergic rhinitis, may have recorded sex differences [51].

### Related factors and characteristics of malocclusion

Based on the above sample characteristics in the present study, a further analysis was conducted to clarify the related factors and characteristics in malocclusion. Crude analysis revealed that ILS (*P* < 0.01), especially severe ILS (*P* < 0.001), and palatine tonsil hypertrophy (*P* < 0.05) recorded significantly higher frequencies, and nail biting (*P* < 0.05) recorded significantly lower frequency, in malocclusion than in normal occlusion. Additionally, the results of adjusted analysis showed a significant association of ILS (*P* < 0.01) and nail biting (*P* < 0.05) with malocclusion. Although ILS was reported to be a factor in malocclusion in some previous studies [27, 52–54], our findings revealed that malocclusion in preschool children was strongly associated with ILS in the cross-sectional study in the present study. Open mouth posture is considered to be associated with a slower pattern of maxillary growth, a narrow maxillary dental arch, and increased facial height [54, 55]. Nail biting was found to be a negative factor for malocclusion in the present study. Additionally, as mentioned above, nail biting was not related to mandibular midline deviation. The effect of nail biting on malocclusion is unclear and is not backed up by clinical or statistically significant evidence [24]. The results of the present study suggest that nail biting may not necessarily be a deleterious habit for the occlusion, but may be a guide for edge-to-edge incisal contact and attrition, resulting in the low prevalence of sagittal and vertical anomalies in preschool childhood.

No significant association with malocclusion was found for nose and throat conditions. Although it is thought that upper airway obstruction can cause mouth breathing and can contribute to a narrow maxillary dental arch and increased facial height, no consensus has yet been reached about an association with malocclusion [54–57]. Further research including accurate diagnosis and assessment of the degree of severity of these conditions is needed.

### Limitations

The present study had some limitations. Our study sample was derived from one kindergarten in a local area of Japan and the sample size was small. Therefore, the data cannot represent the prevalence of malocclusion in all Japanese preschool children. Additionally, because the definition of malocclusion was based on the classification constructed by the Japanese Society of Pediatric Dentistry, we did not include some criteria such as distal steps of the second primary molars and primary canine relationships. Furthermore, it is necessary to consider the intensity, long-term frequency, daily frequency, and duration when examining the impact of oral habits on malocclusion. As for nasal and throat conditions, allergic rhinitis and palatine tonsil hypertrophy may have been under-reported because they were based on a questionnaire. Finally, genetic factors, particularly skeletal patterns, which can significantly influence malocclusion, were not taken into account in the study. However, it should be noted that early detection of oral habits and nasal and throat disease and minimizing their impact are important for proper maxillofacial development in early childhood.

## Conclusions

Within the limitations of the present study, the following conclusions can be drawn. Approximately 60% of Japanese preschool children in the present study exhibited malocclusion, approximately 40% had mandibular midline deviation, and approximately 35% had closed dentitions. 27.8% of children exhibited ILS, and nail biting was the most frequent oral habit with a prevalence of 18.9%. ILS is significantly associated with malocclusion, and nail biting may not necessarily be a deleterious habit for occlusion in Japanese preschool children. We consider that malocclusion in early childhood is strongly affected by environmental factors.

## Data Availability

The dataset used and/or analyzed during the current study available from the corresponding author on reasonable request.

## Abbreviations

ILS: Incompetent lip seal
